# The role of proboscis of the malaria vector mosquito *Anopheles stephensi *in host-seeking behavior

**DOI:** 10.1186/1756-3305-4-10

**Published:** 2011-01-27

**Authors:** Emi Maekawa, Hiroka Aonuma, Bryce Nelson, Aya Yoshimura, Fumio Tokunaga, Shinya Fukumoto, Hirotaka Kanuka

**Affiliations:** 1 National Research Center of Protozoan Diseases, Obihiro University of Agriculture and Veterinary Medicine, Inada-cho, Obihiro, Hokkaido 080-8555, Japan; 2 Department of Biological Sciences, Graduate School of Science, Osaka University, Osaka 560-0043, Japan; 3 Department of Parasitology, National Institute of Infectious Diseases, Tokyo 162-8640, Japan; 4 Donnelly Centre for Cellular and Biomolecular Research, Toronto, Ontario, M5S 3E1, Canada

## Abstract

**Background:**

The proboscis is an essential head appendage in insects that processes gustatory code during food intake, particularly useful considering that blood-sucking arthropods routinely reach vessels under the host skin using this proboscis as a probe.

**Results:**

Here, using an automated device able to quantify CO_2_-activated thermo (35°C)-sensing behavior of the malaria vector *Anopheles stephensi*, we uncovered that the protruding proboscis of mosquitoes contributes unexpectedly to host identification from a distance. Ablation experiments indicated that not only antennae and maxillary palps, but also proboscis were required for the identification of pseudo-thermo targets. Furthermore, the function of the proboscis during this behavior can be segregated from CO_2 _detection required to evoke mosquito activation, suggesting that the proboscis of mosquitoes divide the proboscis into a "thermo-antenna" in addition to a "thermo-probe".

**Conclusions:**

Our findings support an emerging view with a possible role of proboscis as important equipment during host-seeking, and give us an insight into how these appendages likely evolved from a common origin in order to function as antenna organs.

## Background

Mosquitoes transmit pathogens of diseases such as malaria, filariasis, yellow fever, and dengue fever. Malaria, killing nearly one million people annually [1], is caused by infection with parasites of the genus *Plasmodium *that is transmitted by female anopheline mosquitoes. *Anopheles stephensi *mosquitoes are the leading vector of malaria in India, parts of Asia and the Middle East. Despite these control efforts using mosquito nets [2], repellents [3], and insecticide [4,5], malaria remains a leading cause of worldwide morbidity and mortality [1,6]. The rate of contact between vertebrate hosts and mosquito *Anopheles *vectors has long been recognized as a crucial determinant of malaria transmission [7-9], and successful malaria control depends on understanding the interactions between mosquitoes and humans [10-13]. In order for transmission to occur, however, a female mosquito must be able to find potential hosts. In general, it is known that mosquitoes are remarkable for their ability to locate blood meal using human body emanations such as CO_2_, lactic acid, 1-octen-3-ol, and heat acting as strong mosquito attractants [14-16].

For malaria vector preferring warm-blooded animal to cold-blooded animal, heat of the skin is one of the most potent candidate attractants. Blood-feeding kissing bug, *Triatoma infestans*, appears to possess thermoreceptors that enable it to perceive radiant heat from endothermic prey and estimate its temperature [17]. Another blood-feeding insect *Rhodnius prolixus *approaches a thermal source guided solely by its infrared radiation [18]. In 1910, an important stimulatory role for heat emanating from potential hosts was elucidated; when females of *Aedes *(*Stegomyia*) *scutellaris *were placed in a loose gauze bag with a test tube containing hot water held nearby, the insects became restless upon exposure to the hot air [19]. In 1918, it was reported that a glass plate heated to just one degree (F°) above human body temperature was sufficient for attraction of mosquitoes [20]. By the early 1950's it was suggested that heat was the prime factor in attracting and inducing female mosquitoes to probe host skin [21]. In fact, the hands of warm-skinned Caucasian individuals were found to be more attractive to *Ae. aegypti *than cool-skinned individuals, and an artificially cooled hand or body was much less attractive than a normal one [22,23]. Recently, evidence for thermo-sensitive sensilla on mosquito appendages has been uncovered [24]. It was reported that activation of a transient receptor potential (TRPA1), one of the ion channels involved in various types of sensory reception, including thermoreception, chemoreception, mechanoreception, and photoreception, is caused by an increase in temperature from 25 to 37°C in *Anopheles gambiae *[24]. However, an organ (appendage) contributing to heat sensing in host-seeking behavior still remains to be elucidated.

Here we established an automatic recording device to quantify CO_2_-activated thermo (35°C)-sensing behavior of mosquito. In this study, we present the first evidence that the mosquito proboscis participates in thermo-sensing in order to locate the target during the host-seeking process. Our results suggest that each appendage in mosquito head (antenna, maxillary palp, and proboscis) shares roles in sensing attractant and stimulant factors, leading to the capture of host.

## Results and Discussion

### The mosquito proboscis is involved in host recognition

**Figure 1:**
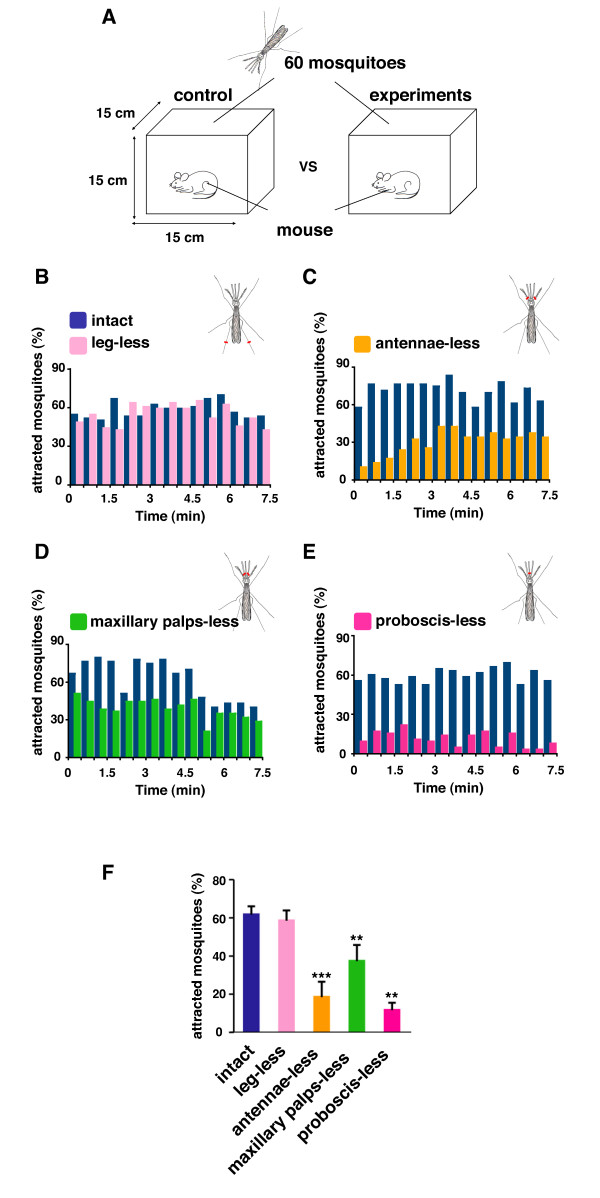
**The mosquito proboscis is involved in host recognition**. (A) Schematic of host recognition assay (see Materials and Methods). 120 female mosquitoes were divided into two groups, intact (control) and appendage-ablated (experiment). Mice were placed individually into indicated cages and monitored simultaneously by counting the number of mosquitoes sucking blood. (B-F) To determine the contribution of each appendage to host recognition, each group of female mosquitoes lacking specific body parts (leg, antennae, maxillary palps, or proboscis) was prepared for the assay shown in (A). The proportion of mosquitoes touching down on mice was calculated every 0.5 min for 7 min. Blue bars represent controls while other colored bars represent experimental groups. Note that a mosquito's ability to recognize a host is clearly reduced upon removal of the proboscis (E and F). (F) **p < 0.005, ***p < 0.001, t-test; mean ± SD, n = 4.

**Figure 2:**
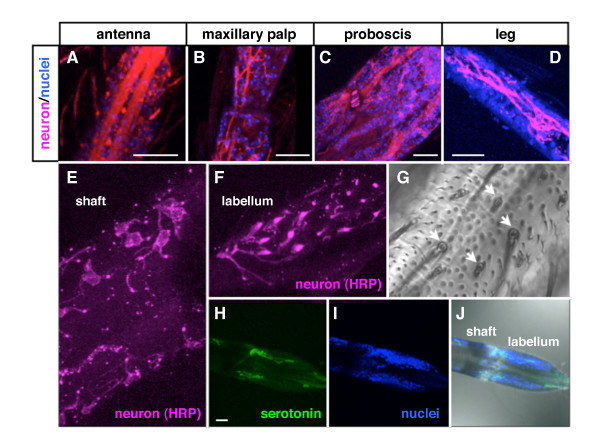
**Histological similarity between proboscis and other appendages**. (A-D) Distribution of neurons in four different types of mosquito appendages. Panels represent the merged images of immunostaining of antenna (A), maxillary palps (B), proboscis (C), and leg (D) (blue: nuclei, magenta: HRP-positive neuron). Note the similar large bundles of nerve fibers visualized in each appendage. (E-J) A variety of neurons in mosquitoes' proboscis. Panels represent the localization of HRP-positive neurons (magenta) in the shaft (E) and labellum (F) of the proboscis. The proboscis also contains serotonin-positive neurons (blue: nuclei, green: serotonin) (H-J). Note that there are many sensilla (extra sensory organs) distributed throughout the proboscis (G). All bars: 25 μm.

Like most dipterans, the head of adult mosquitoes is equipped with three types of appendages: antennae, maxillary palps, and proboscis. Previous experiments demonstrated that antennae and maxillary palps were both involved in host detection by a series of experiments in which each appendage was surgically removed from the mosquito head [25-30]. In order to revisit the roles of macro-type extrasensory organs of *Anopheles stephensi*, a major pathogen vector species, mosquitoes with surgically ablated appendages were examined for the ability to recognize mice as a blood-source (Figure 1A). To exclude the possibility of physical damage affecting the behavior of mosquitoes hind legs were removed as a control (Figure 1B and 1F: leg-less vs. intact, N.S.). Consistent with previous reports, removal of either antennae or maxillary palps led to a drastic reduction in the mosquitoes' ability to locate a host (Figure 1C, D and 1F: antennae-less vs. intact, *p *< 0.001; maxillary palps-less vs. intact, *p *< 0.005), suggesting that these organs are equipped to find the host before landing. Surprisingly, ablation of the entire proboscis drastically reduced the mosquitoes' ability to detect a host (Figure 1E and 1F: proboscis-less vs. intact, *p *< 0.005) raising the intriguing possibility that the proboscis might not just function as a tool for food intake but might also serve as an antenna for target recognition. Indeed, the mosquito proboscis shares many structural features with other canonical antenna such as the maxillary palps including large bundles of nerve fibers as visualized by an antibody to the pan-neuronal marker HRP (Figure 2A-D). The proboscis, in particular, had a variety of neurons in the shaft and labellum that corresponded to the position of sensory hairs (Figure 2E-G), in addition to large numbers of serotonin neurons (Figure 2H-J). Given that all appendages on insect heads are variations of the same homologous structure extended from each body segment [31] and that the forelegs of at least one hematophagous tick are used as antenna to recognize distant hosts [32] we propose that the proboscis of mosquitoes might possess functional similarity with other typical sensory organs such as the antenna and maxillary palps.

### Automated-device for quantifying the selected host-seeking behavior of mosquitoes

**Figure 3:**
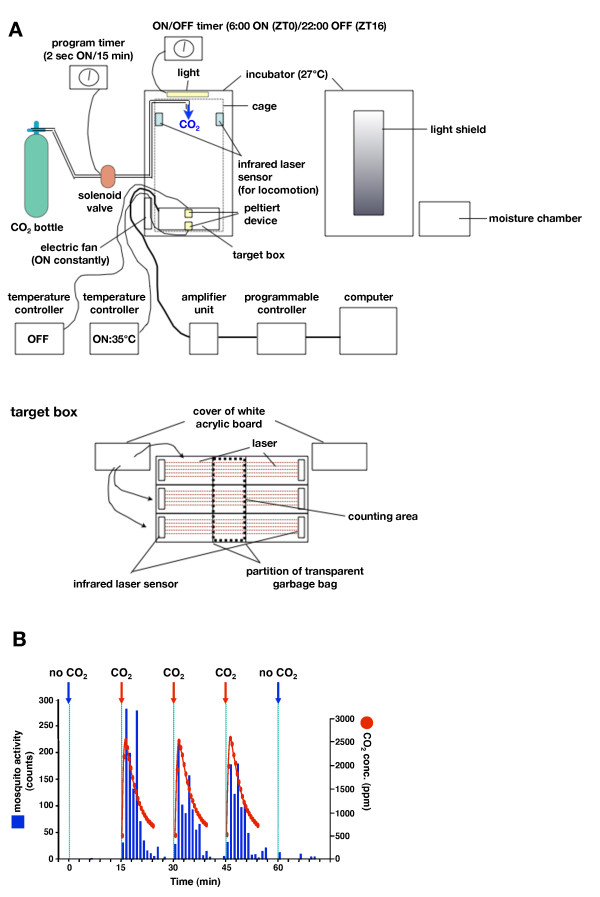
**Automated-recording device for quantifying the selected host-seeking behavior**. (A) Schematic view of the recording device to quantify mosquito activity patterns (see Materials and Methods). (B) An example of the selected host-seeking behavior of 60 female mosquitoes monitored for a short period (75 min) by automated-device shown in (A). CO_2 _was delivered for 2 sec at 15 min, 30 min, and 45 min (arrow). Note that female mosquitoes show "touch-down" behavior (blue) on the Peltier plate in response to the change of CO_2 _concentration (red).

**Figure 4:**
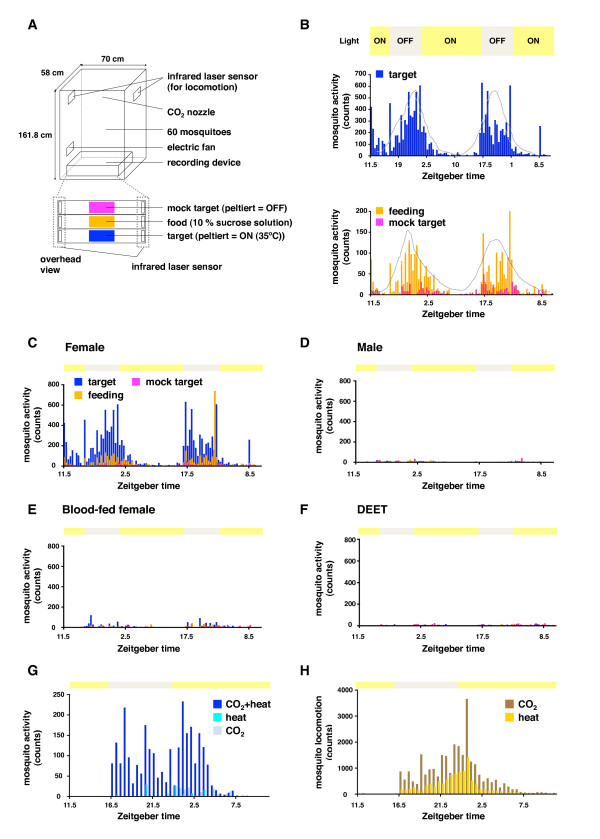
**Mosquito host-seeking behavior is reproduced in the artificial environment**. (A) Schematic view of a mosquito cage containing a recording device. (B) A typical pattern of selected host-seeking behavior of 60 female mosquitoes monitored for 48 h by the automated-device shown in (A). CO_2 _is delivered intermittently (2 sec every 15 min) during the 48 hours assay. Upper crossbar indicates light (yellow)-dark (gray) timer controlled conditions (6:00 ON; 22:00 OFF). (C-F) Patterns of selected host-seeking behavior (48 h) of females (C), males (D), blood-fed females (E), and females served with target Peltier covered with DEET (F). Note that only non blood-fed female mosquitoes show the selected host-seeking behavior. Mosquito behavior is represented as: host-seeking (blue), sugar-feeding (orange), and background behavior (magenta). (G) Both CO_2 _and heat are essential for activation of the selected host-seeking behavior (24 h). The behavioral assay was performed for 24 hours under each condition with CO_2 _only (light blue), heat only (light green), and CO_2 _+ heat (blue), respectively. (H) CO_2_-activated simple locomotion activity of female mosquitoes (24 h). Note that female mosquitoes show higher locomotion activity induced by CO_2 _(dark orange) than by heat (light orange).

To verify a concealed role for the mosquito proboscis during host recognition, we created an automated device able to quantify selected host-seeking behavior (Figure 3A and 4A). This device is able to simultaneously monitor three independent mosquito behaviors (touch-down on target (1) and mock (2), and sugar-feeding (3)), in addition to locomotion activity (free flying) for 24 consecutive hours. Sixty mosquitoes at a time were allowed to fly freely in the space for a week with continuous monitoring. We integrated a carbon dioxide (CO_2_) and heat source into the device in order to elicit selected host-seeking behavior since both factors are observed commonly in all warm-blooded animal hosts. The Peltier (pseudo-target), controlled at 35°C to mimic body temperature of humans, was placed together with an infrared laser sensor at the bottom of the cage, while 2 second CO_2 _bursts were delivered from the upper part of the cage every 15 minutes. The device scores "one count" when a mosquito crosses the infrared laser to land on or to leave from the target. Another Peltier without heating was also set in the device to monitor mosquito behavior against the mock target (background behavior) in addition to quantifying sugar-feeding behavior as evaluating physical condition of mosquitoes. In this monitoring device, female mosquitoes showed repeated "touch-down" behavior (mimicking landing on the host surface) on the heated Peltier in response to CO_2 _(Figure 3B and 4B). Consistent with nocturnal blood feeding patterns of wild *A. gambiae *in endemic areas the majority of CO_2_-activated target-capturing behavior was observed approximately between 22:00-6:00 [33] (Figure 4B and 4C). In order to confirm that the observed host-seeking behavior mimicked that of wild mosquitoes we tested both male and blood-fed female in the device; host-seeking behavior is specific for blood-fasted female mosquitoes and drastically disappears after blood-feeding [34]. In addition, we assayed blood-fasted female mosquitoes using a Peltier coated with N,N-Diethyl-m-toluamide (DEET), the most effective and commonly-used mosquito repellent. In all these experiments the host-seeking behavior was nearly cancelled (Figure 4D-F), indicating that the host-seeking behavior of mosquitoes in the field was well reproduced in the artificial environment.

### Segregation of CO_2 _and heat-sensing behavior mediated by mosquito proboscis

**Figure 5:**
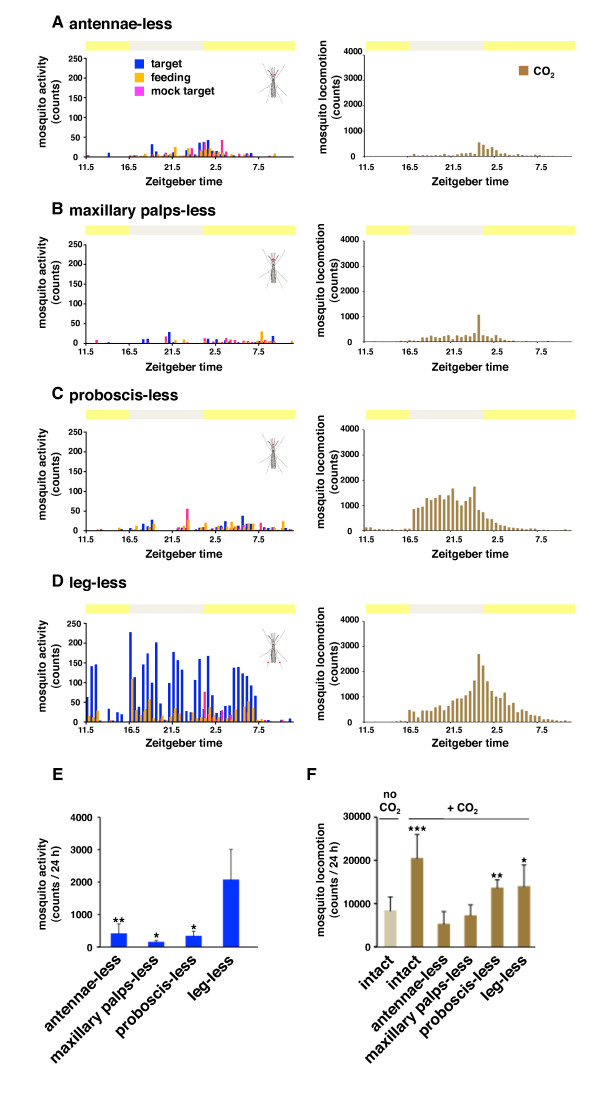
**Segregation of CO**_**2 **_**and heat-sensing behavior mediated by mosquito proboscis**. (A-D) Effect of appendage-ablations on the selected host-seeking behavior (left column) and CO_2_-activated locomotion (right column). Behaviors of each group of 60 female mosquitoes lacking antenna (A), maxillary palps (B), proboscis (C), or leg (D) were monitored for 24 h by the automated-device. CO_2 _was delivered intermittently (2 sec every 15 min) during the 24 hours assay. Each graph shows selected host-seeking (blue), sugar-feeding (orange), background (magenta), and CO_2_-activated locomotion behavior (dark orange). (E) Total activity counts of selected host-seeking behavior during the 24 hours assay. Note that antenna-, maxillary palps-, and proboscis-less mosquitoes show significantly-reduced activity compared to the leg-less mosquitoes (**p < 0.01 and *p < 0.05, t-test; mean ± SD, n = 5). (F) Total activity counts of CO_2_-activated locomotion during the 24 hours assay. Note that antenna- and maxillary palps-less, but not proboscis-less mosquitoes show significantly-reduced locomotion compared to the intact mosquitoes without CO_2 _(***p < 0.005, **p < 0.01 and *p < 0.05, t-test; mean ± SD, n = 5).

We next examined the possible contribution of these head appendages to each individual host-seeking behavior. Ablation of either maxillary palps or antennae clearly disturbed the CO_2_-activated seeking of heated targets (Figure 5A and 5B, left column) (Figure 5E: antennae-less vs. leg-less, *p *< 0.01; maxillary palps-less vs. leg-less, *p *< 0.05), consistent with both the results of the host-recognition assay (Figure 1B-D, and 1F) and previous reports [25-30]. Likewise, the proboscis-less mosquitoes also displayed an inability to seek a heated target (Figure 5C, left) (Figure 5E: proboscis-less vs. leg-less, *p *< 0.05), strongly suggesting that the proboscis plays a key role during either CO_2 _or heat recognition. To tease out the precise function of the proboscis, we then separately quantified CO_2_-activated locomotion activity using additional infrared laser sensors placed at the top of the cage (Figure 3A and 4A). Consistent with previous reports showing that probing behavior in *Aedes aegypti *requires an increase in CO_2 _content, either via a host or artificially [35], and *Ae. aegypti *are sensitized to human skin odors upon CO_2 _exposure [36], we found that both CO_2 _and heat were indispensable for initiating and completing host-seeking behavior (Figure 3B and 4G). Importantly, we also observed that female mosquitoes exhibited high locomotion activity upon induction by CO_2 _alone without subsequent pseudo-target recognition (Figure 4H and 5F: intact with CO_2 _vs. intact without CO_2_, *p *< 0.005). As a result, while the CO_2_-induced locomotion of antenna- or maxillary palp-less mosquitoes was significantly reduced (Figure 5A and B, right column) (Figure 5F: antennae-less with CO_2 _vs. intact without CO_2_, N.S., maxillary palps-less with CO_2 _vs. intact without CO_2_, N.S.), proboscis-less mosquitoes retained CO_2 _sensitivity to a level comparable to leg-less mosquitoes (Figure 5C and 5D, right) (Figure 5F: proboscis-less with CO_2 _vs. intact without CO_2_, *p *< 0.01; leg-less with CO_2 _vs. intact without CO_2_, *p *< 0.05). Taken together, these results imply that the proboscis of mosquitoes plays a distinct role specifically during the thermo-sensing stage of host-seeking.

### Mosquito TRPA1 is a candidate thermo-sensing protein for host-seeking behavior

**Figure 6:**
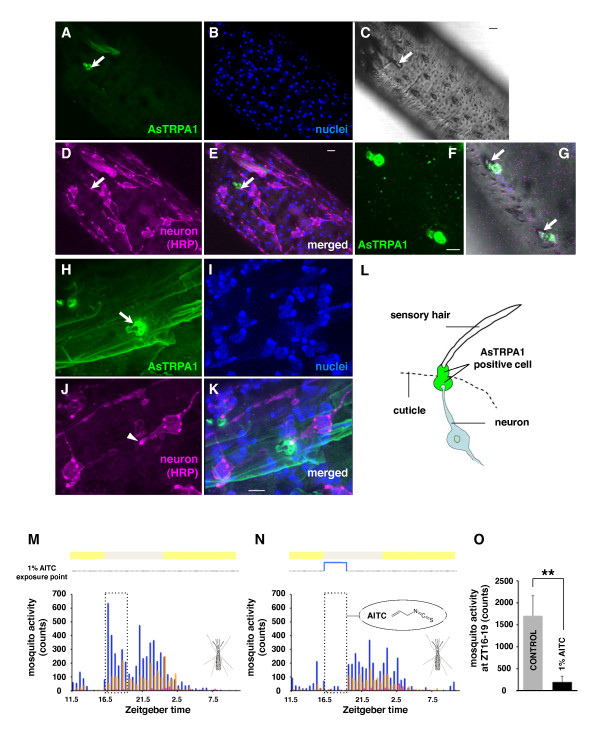
**Mosquito TRPA1 is a candidate thermo-sensing protein for host-seeking behavior**. (A-L) AsTRPA1 (+) cells (green) are present along the whole proboscis and in association with sensory neurons (magenta: HRP-positive neuron, D and J). Note that some sensilla contain AsTRPA1 (+) signals (A, F, and H). All bars: 5 μm. (M-O) TRPA1-activating agent (AITC) disturbs the selected host-seeking behavior. Female mosquitoes were exposed to 1% (v/v) AITC at ZT16-19. Note that volatile AITC reduces the selected host-seeking behavior compared to controls (**p < 0.01, t-test; mean ± SD, n = 5).

In both vertebrates and invertebrates, temperature sensation is mediated through activation of TRP channels able to detect heat or cold [37-39]. Recently, it was reported that the TRPA1, a TRP family channel, functions as an infrared detector in the pit viper during prey recognition [40]. Conservation of this function has been demonstrated when it was shown that *A. gambiae *TRPA1, AgTRPA1, conferred responses to temperature increases when functionally expressed in *Xenopus *oocytes [24]. To examine a putative role for TRPA1 in the mosquito proboscis, we produced a polyclonal antibody to *A. stephensi *TRPA1 peptides and probed for protein presence in the proboscis. Indeed, AsTRPA1-expressing cells were observed along whole proboscis with localization just beneath sensilla that are in association with sensory neurons in both female, male, and blood-fed female (Figure 6A-G and data not shown). In addition, we also found that the AsTRPA1-expressing cells closely located at the nerve terminal and base of the sensilla (Figure 6H-K), suggesting that AsTRPA1 is not expressed in neurons, at least in a few accessory cells such as trichogen, tormogen, and/or thecogen cells which form shaft, sheath, and socket of insect sensilla (Figure 6L). We also used the volatile reactive electrophile allyl isothiocyanate (AITC) that covalently binds and activates *A. gambiae *TRPA1 when expressed in *Xenopus *oocytes [41]. When mosquitoes were exposed to 1% AITC, the selected host-seeking behavior was drastically reduced before gradual recovery upon chemical withdrawal (Figure 6M and 6N) (Figure 6O: AITC (+) vs. AITC (-), *p *< 0.01), suggesting that heat-sensing via AsTRPA1 molecules was disrupted by AITC. AsTRPA1 is, therefore, a candidate molecule for the transfer of heat information from the host to the chemosensory neurons following channel activation.

**Figure 7:**
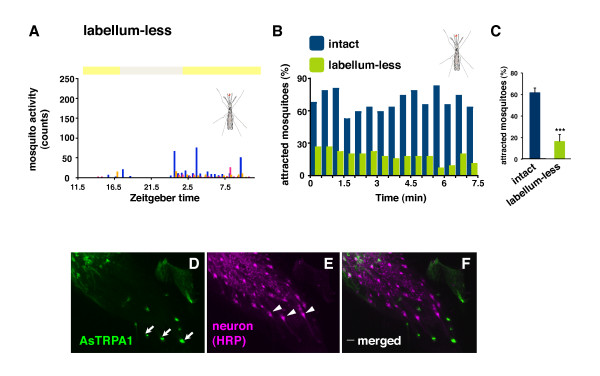
**Labellum of the mosquito proboscis contributes to host-seeking behavior**. (A-C) A possible role for the mosquito labellum as a thermo-detector. Note that the ability of labellum-less mosquito to recognize pseudo-targets (A) and real hosts (B) is reduced (C) (***p < 0.001, t-test; mean ± SD, n = 4). (D-F) AsTRPA1 is expressed on the labellum. Scale bars: 5 μm.

In order to determine the "thermo-detector" portion of the proboscis, we cut the distal tip of the proboscis, known as the labellum that contains gustatory sensilla and likely serves as the functional equivalent of the mammalian tongue. As a result, the ability of labellum-less mosquitoes to recognize both pseudo-targets (Figure 7A) and real hosts (Figure 7B) was extremely reduced compared to control mosquitoes (Figuer 7C: labellum-less vs. intact, *p *< 0.001). In addition, we observed a set of AsTRPA1-expressing cells at nerve terminal and base of sensilla (Figure 7D-F). Given that the labellum contains abundant sensilla responsive to a variety of odorants and other stimuli through odorant and gustatory receptors [42], our results provide important insight into host recognition by vector mosquitoes suggesting that the protruding proboscis may work as an alternative antenna in addition to the canonical antenna and maxillary palps.

### Evolutionally diversion of thermo-probe to a role of a thermo-antenna

Despite specializations into multiple appendage types, such as antennae, maxillary palps, legs, and proboscis, modern insect appendages are considered to be serially homologous structures that retain anatomical and developmental aspects of their common evolutionary origin [31]. Alternatively, there have been considerable studies supporting our hypothesis that any type of appendage can evolve to take on functions similar to antennae during the course of evolution. Firstly, in addition to widespread expression of odorant receptors (ORs) in the olfactory organs such as antennae and maxillary palps of the vector mosquito species, *Anopheles *and *Aedes*, OR7, an obligatory partner protein of a variable odorant-binding OR required to create a functional ion channel, is commonly expressed in the proboscis [42]. Secondly, the oviposition behavior of butterflies is elicited by recognition of plant compounds via receptors in the tarsus of the foreleg [43]. Thirdly, tick forelegs are known as antennae necessary for the recognition of distant hosts using the Haller's organ, a sensory structure containing sensilla on the dorsal surface of the leg [32]. Fourthly, a previous observation that olfactory receptor neurons for CO_2 _detection can relocate from antennae to maxillary palps in *Drosophila *suggests antenna-like appendages have flexibility to carry out their sensory functions [44]. With respect to evolution, six-legged ancestors came out of the water and onto dry land over 400 million years ago, whereas mammals, the current targets of mosquitoes, first appeared in the fossil record about 230 million yeas ago. Presumably, prior to the appearance of warm-blooded animal such as mammals, mosquitoes must have adopted other targets such as reptiles, amphibians, and fish and would not have had pressure to develop a prototype thermo-antenna. In contrast, it is possible that the origin of the thermo-probe was in response to dangers such as fire. In order to precisely discriminate between warm-blooded animals and fire, the thermo-probe, previously functioning for emergencies might have been diverted or switched to a role of a thermo-antenna.

## Conclusions

We have provided the first evidence that a mosquito proboscis can function as a thermo-sensory organ during orientation behavior with implications for prospective control purposes through genetic manipulation of host preference. Considering the role of the proboscis as a thermo-antenna during host-seeking, our discovery may provide a novel blueprint for mosquito sensory systems that is likely to influence strategies for vector control including the development of effective insect traps.

## Materials and methods

### Mosquito rearing and maintenance

A wild type strain of laboratory-reared *Anopheles stephensi *was used throughout this study (a gift from Dr. Y. Chinzei). Adult females and males were kept together in mesh nylon cages (30 cm × 30 cm × 30 cm) under the following conditions: 27°C; 80% R.H.; 12 h:12 h = L:D photoperiod. These mosquitoes had constant access to a 10% sucrose solution on filter paper. Eggs laid on wet filter papers were transferred to water trays. Larvae were fed carp food (Hikari; Kyorin corporation). 4- to 10-day old females were used in all experiments in this report.

### Host recognition assay

120 female mosquitoes were divided into two groups (control and experimental group). The experimental group was prepared as follows: each set of appendages (antennae, maxillary palps, proboscis, or hind legs) was removed using sharpened tweezers (DUMONT DUMOXEL 5) under CO_2 _anesthesia. Treatment had negligible impact on survival rates of mosquitoes and flying activity during host seeking behavior (data not shown). The control group was anesthetized with CO_2 _in the same manner as the experimental group. The mosquitoes of each group were put into a small cage (15 cm × 15 cm × 15 cm) and kept overnight under normal condition as described above. An anesthetized female mouse (BALB/c: 5-7 weeks old, CLEA Japan, Inc.) was placed into each cage at the same time and pictures of both cages were recorded from above by a high-speed digital camera (EX-F1, CASIO) every 30 sec for 420 sec in order to count the number of mosquitoes settling or landing on the mouse.

### Automatic recording device for quantifying mosquito behavior

The recording device was composed basically of three infrared laser sensors (LV-H300), amplifiers (LV-51M), a programmable controller unit (KV-700), and monitoring software (Keyence Corporation) (Figure 3A and 4A). The infrared laser sensor was composed of a laser releaser and acceptor kept approximately 30 cm apart. These 3 sensors were placed in parallel at the bottom of a large nylon mesh and metal frame cage (70 cm × 58 cm × 161.8 cm) set in the incubator (MIR-253, SANYO) maintained under a photoperiod of 16 h:8 h (L:D), 27°C, and >60% RH. In order to measure three kinds of mosquito behaviors (host-seeking, background, and sugar-feeding behavior), a heated Peltier (35°C), a powered-off (cool) Peltier, and a small conical flask with 10% sucrose solution (respectively) were placed at the center of each sensor. The surface of each Peltier plate was covered with white paper so that the plate becomes visually imperceptible to mosquitoes. The temperature of the Peltier plates (VICS, Tokyo, Japan) was regulated by the controller (VPE-10, VICS). To measure CO_2_-activated simple locomotion, another 4 sets of infrared laser sensors were placed in parallel at the upper space of the cage. CO_2 _release (2 sec at 15 min intervals) from a nozzle at the top of the cage was controlled by a solenoid valve (FSD-0408C, Flon Industry Co., Ltd., Tokyo, Japan) equipped with intermittent timer (FT-022, TGK, Tokyo, Japan). The air inside the cage was constantly ventilated by an electric fan (VFP-8CS3, TOSHIBA) located in the lower side of the incubator. The concentration of CO_2 _inside the cage, measured using a CO_2 _detector (TECH-JAM Co., Ltd., Osaka, Japan), was approximately 5 times higher than background upon the CO_2 _release before reducing gradually within 15 minutes (Figure 3B). For all experiments, mosquitoes were first put into the cage containing the recording device and allowed overnight acclimation. Next day mosquitoes were collected in small vials for each treatment. Blood-fed female mosquitoes were prepared via sucking blood of mice for 1-2 h. 60 mosquitoes were then collected in small vials again, anesthetized with CO_2_, and transferred to the cage 2-3 h before each experiment.

### Immunohistochemistry

Immunostaining was carried out as previously reported [45] with some modifications. Briefly, after decapitation into 4% PFA in PBSTx (PBS with 0.25% Triton X-100), each appendage (antennae, maxillary palps, proboscis, and legs) was cut into small pieces by using a scalpel blade (Feather, NO.11) or ultrasonic homogenizer (VP-300; Taitec) for a few seconds. The following antibodies and fluorescent material were used; rabbit anti-horseradish peroxidase antibody (Jackson Laboratories), goat anti-horseradish peroxidase (Cappel (#55970)), rabbit anti-serotonin antibody (SIGMA (S5545)), goat anti-mouse IgG-Alexa 488 (invitrogen), donkey anti-rabbit IgG-Alexa 488 (invitrogen), donkey anti-goat IgG-Alexa 568 (invitrogen), donkey anti-mouse IgG-Alexa 647 (invitrogen). Polyclonal anti-AsTRPA1 antiserum was developed in rabbits using a synthetic peptide corresponding to amino acids (GNVPLHSAVHGGDIC) of *A. stephensi *TRPA1, conjugated to KLH via an N-terminal added cysteine residue as an immunogen. The antiserum was purified using HiTrap Protein G HP 1 ml (GE Healthcare). Preimmune serum or absent primary antibody was used to confirm the specificity of the AsTRPA1 antibody. All antibodies described above were diluted 1:1000 in 5% goat or donkey serum. Nuclei were labeled using TO-PRO-3 (1:300, invitrogen). All fluorescent images were examined using a TCS SP5 confocal microscopy (Leica).

## Competing interests

The authors declare that they have no competing interests.

## Authors' contributions

EM conceived the study, performed the experiments, and wrote the manuscript. HA and AY helped collection of mosquitoes. BN clarified the manuscript. FT and SF conceived and supervised the study. HK supervised the study. All authors read and approved the final manuscript.
